# Shifting the paradigm: engaging multicellular networks for cancer therapy

**DOI:** 10.1186/s12967-024-05043-8

**Published:** 2024-03-12

**Authors:** Joyce Hu, Paolo Ascierto, Alessandra Cesano, Volker Herrmann, Francesco M. Marincola

**Affiliations:** 1 Sonata Therapeutics, Watertown, MA 02472 USA; 2 Cancer Immunotherapy and Innovative Therapy, National Tumor Institute, Fondazione G. Pascale, 80131 Naples, Italy; 3 ESSA Pharmaceuticals, South San Francisco, CA 94080 USA

## Abstract

Most anti-cancer modalities are designed to directly kill cancer cells deploying mechanisms of action (MOAs) centered on the presence of a precise target on cancer cells. The efficacy of these approaches is limited because the rapidly evolving genetics of neoplasia swiftly circumvents the MOA generating therapy-resistant cancer cell clones. Other modalities engage endogenous anti-cancer mechanisms by activating the multi-cellular network (MCN) surrounding neoplastic cells in the tumor microenvironment (TME). These modalities hold a better chance of success because they activate numerous types of immune effector cells that deploy distinct cytotoxic MOAs. This in turn decreases the chance of developing treatment-resistance. Engagement of the MCN can be attained through activation of immune effector cells that in turn kill cancer cells or when direct cancer killing is complemented by the production of proinflammatory factors that secondarily recruit and activate immune effector cells. For instance, adoptive cell therapy (ACT) supplements cancer cell killing with the release of homeostatic and pro-inflammatory cytokines by the immune cells and damage associated molecular patterns (DAMPs) by dying cancer cells. The latter phenomenon, referred to as immunogenic cell death (ICD), results in an exponential escalation of anti-cancer MOAs at the tumor site. Other approaches can also induce exponential cancer killing by engaging the MCN of the TME through the release of DAMPs and additional pro-inflammatory factors by dying cancer cells. In this commentary, we will review the basic principles that support emerging paradigms likely to significantly improve the efficacy of anti-cancer therapy.

## Direct (primary) versus indirect (secondary) cancer cell killing

**Fig. 1:**
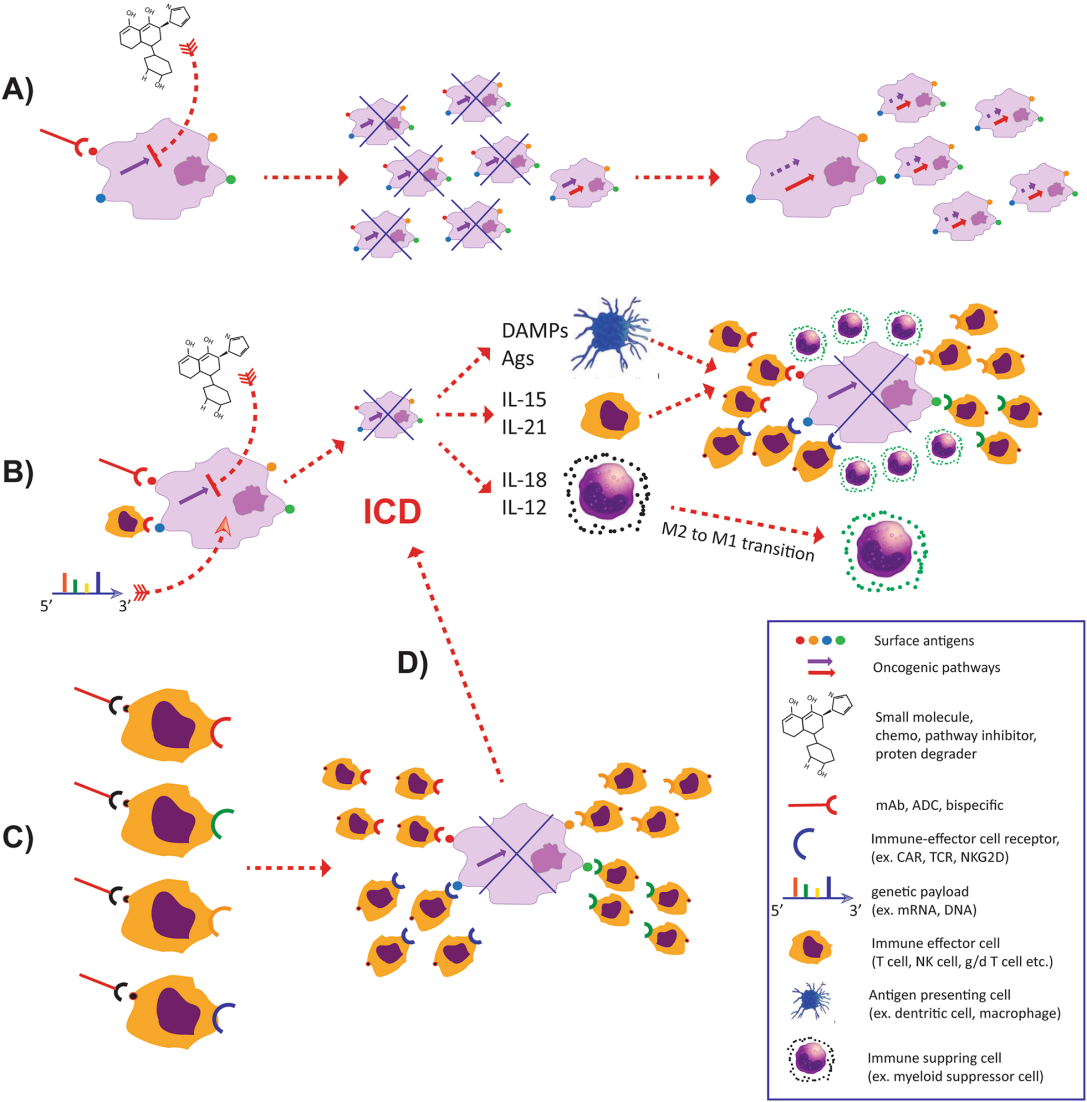
– Direct vs indirect models for cancer cell elimination. **A** Linear model of direct cancer cell killing; by targeting a specific attribute of cancer cells that differentiate them from benign cells exemplified here by a traditional small molecule directed against a cancer-driving pathway or a biological binder directed against an antigen that can be recognized on the surface of cancer cells. Cancer elimination depends solely on the linear relationship between the mechanism of action (MOA) and the relevance of its target. Cancer cells that eliminate the target become resistant to therapy. **B** Exponential model of direct cancer cell killing; Therapies including ‘smart’ small molecules, biologics such as ADCs, CAR T cells and TILs, and genetic payloads can induce cancer cell death and the release of tumor-associated antigens (Ags) and damage associated molecular factors (DAMPs) perceived as abnormal by the multicellular network (MCN) in the tumor microenvironment (TME), thus initiating a mechanism referred to as immunogenic cell death (ICD). This elicits the recruitment of immune cells in the TME that can lead to further cancer killing dependent on additional MOAs distinct from the original one. Complex therapeutics such as activated immune effector cells delivered through adoptive cell therapy (ACT), can add the secretion of homeostatic cytokines such as interleukin (IL)-2 that sustain their persistence and proinflammatory cytokines such as interferon (IFN)-$\gamma$ and tumor necrosis factor (TNF)-α that can redirect an immune suppressive cellular network into one hostile to cancer cell survival. This can further escalated by the delivery of genetic information that induces the production of genes not normally produced by immune effector cells such as IL-15, IL-12, IL-18, that further amplify the anti-cancer cell reaction by recruiting additional immune effector mechanisms that employ additional MOAs. **C** Linear model of indirect cancer killing: the therapeutic targets a specific function of a cellular component of the multicellular network in the TME through a single MOA. Here exemplified by a mAb targeting a checkpoint receptor such as programmed cell death protein 1 (PD1) present on the surface of immune effector cells, in particular CD8^+^ antigen-specific T cells, allows for proliferation and stabilization of  function. Since the resident CD8^+^ T cells recognize different Ags, the anti-cancer response is amplified by recognizing multiple targets relevant to the specific TME compared to biologics that target a predetermined Ags. **D** Conditional exponential model of indirect cancer killing: “*if*” successful indirect cancer killing can ignite the exponential model described in B). However, the indirect approach depends on the presence of PD1 expressing CD8^+^ T cells in the TME and the weight that PD1 plays over other mechanism of immune suppression. This concept applies to all methods targeting a benign component of the TME based on a single MOA

Most anti-cancer modalities like chemotherapy, pathway inhibitors and biologics such as monoclonal antibodies or antibody–drug conjugates (ADCs) are designed to exploit a single or at most a few mechanism(s) of action (MOA) for direct, target-specific killing of cancer cells (Fig. 1A). In the context of rapidly evolving cancer genetics, this linear approach based on narrow MOAs frequently leads to the development of resistant subclones through loss of target expression or circumvention of its biological relevance.

Nevertheless, direct killing offers several advantages: (1) it acts directly on neoplastic cells, which are the only authentic cancer-specific entity; (2) the therapeutic index can be accurately calculated based on the differential effects of the MOA between neoplastic and benign tissues; (3) it is not conditional to the presence and functional status of surrounding benign cells; (4) it sheds tumor associated antigens (Ags), and (5) bears the potential of releasing pro-inflammatory signals to initiate, in the tumor microenvironment (TME) and nowhere else, an exponential chain reaction redirecting the MCN from a cancer cell-nurturing phenotype to one hostile to its survival [1, 2] (Fig. 1B). The vicarious modulation of components of the multi-cellular network (MCN) in the TME can be achieved by small molecules that activate multiple anti-tumoral mechanisms [3] that we refer to as ‘smart’ small molecules, pathway inhibitors [4], biologics such as ADCs [5, 6], chimeric antigen receptors (CAR) T cells or tumor infiltrating lymphocytes (TIL) [7] and genetic engineering modalities such as polycistronic mRNA or DNA constructs that can simultaneously deliver pleiotropic payloads [8]. Such modalities can result in amplification of anti-cancer responses through the secondary engagement of endogenous immune effector functions.

Other modalities primarily target benign components of the MCN of the TME such as angiogenic, stromal, myeloid, and effector immune cells to indirectly promote cancer eradication (Fig. 1C) with the ultimate goal, “*if*” successful, to induce an exponential amplification of endogenous anti-cancer mechanisms (Fig. 1D). This strategy stands on the premise that the targeted benign cells are (1) present in the TME and (2) differ from their counterpart in the rest of a healthy organism. However, this is not always the case.

The composition of the MCN in the TME varies from cancer to cancer and targeted cells may or may not be present. This is well exemplified by the dramatic differences in the presence and localization of CD8^+^ T cells in the TME demarcating distinct cancer immunophenotypes [9]. Such variability is observable for most benign components of the multicellular network sustaining the neoplastic formation [10].

Moreover, the presumption that benign cells in the TME are different from their counterparts in a healthy organism does not always hold true. Differences are attributed to the fact that cancers behave as chronically inflamed neoformations that differentiate benign cells in the TME by stimulating reparative and immune functions otherwise absent elsewhere in normal tissues [1, 11–13]. But smoldering chronic inflammatory or infectious processes can occur in seemingly healthy individuals and become the target of treatment-induced, immune-related adverse events as is well exemplified by the erratic occurrence of toxicities during checkpoint inhibitor (CPI) therapy [14].

Finally, indirect cancer cell targeting hinges on the relevance of a single component of the multicellular network of the TME. For instance, CPI therapy against programmed cell death protein 1 (PD1), or cytotoxic T-lymphocyte associated protein 4 (CTLA4) targets predominantly T cells [15–17], while inhibition of the CD47-signal-regulatory protein α (SIRPα) axis focuses on the interactions between cancer cells and macrophages [18]. The narrow scope of each therapy results in occasional efficacy because it addresses only one of the multiple immune regulatory mechanisms that allow the survival of cancer in the immune competent host [19]. In this scenario, responses are observed only when the targeted mechanism is dominant over other ones.

In summary, indirect cancer killing is based on the assumption that (1) benign immune effector cells are present in the TME (which is not necessarily the case specifically for ‘immune desert’ tumors) [20]; (2) the cells differ from their counterparts in non-neoplastic tissues (which is not the case when chronic inflammatory processes assimilate benign tissues to neoplastic formations) [12]; (3) the MOA targeting their modulation is the dominant determinant of the immune biology of individual cancers (which is not always the case due to the complexity of the mechanisms of compensatory immune resistance) [19]. Thus, we believe that direct targeting is most likely to address specificity and efficacy in most conditions.

### The best chance is the first

Because of its genetic instability, cancer is a movable target. Thus, for any anti-cancer therapy, the best chance of success comes at the first cycle of therapy before the neoplasia has the opportunity to develop therapy-resistant clones. Unless the treatment can eliminate all cancer cells at the first round, likelihood of recurrence through expansion of therapy-resistant clones is high and long-term benefit unlikely. This is because, cancer is a fast-track evolutionary process that rapidly adapts to environmental pressure by making the target irrelevant either by losing its expression as is the case for tumor-associated antigens (Ags) [21] or by circumventing its oncogenic properties in the case of pathway inhibitors [4] by acquiring alternative oncogenic mechanisms (Fig. 1A). These escape mechanisms occur by chance depending on the frequency in which genomic alterations accumulate in genetically unstable cells. Moreover, since cancer cell populations are inherently heterogenous, the pre-existence of therapy-resistant cell clones is high. Thus, the narrower the MOA, the higher the chance that cancer can circumvent it. Therefore, the best chance is to complement direct killing of cancer cells with the secondary induction of a myriad of anti-cancer MOAs deployed by activated immune effector cells of the MCN.

Combination therapies with multiple drugs apply two or more anti-cancer MOA with the purpose of decreasing the chance of developing resistance. Adoptive cell transfer (ACT) with immune effector cells, while directly killing cancer cells, expands the MOA by producing homeostatic cytokines and pro-inflammatory factors that in turn recruit and activate endogenous natural killer cells, $\gamma$-$\delta$ T cells, neutrophils and macrophages and other α–β T cells whose cytotoxic properties are directed against different targets on the surface of cancer cells [22–26]. Similarly, chemotherapeutics, pathway inhibitors or biologics that, beyond direct cancer cell killing, can redirect the MCN of the TME through the release by the dying cancer cells of proinflammatory signals, vicariously expand the original MOA and are, therefore, more likely to induce complete eradication of cancer and long-term remissions [2, 3, 5, 6, 27–29] (Fig. 1B).

Thus, the ideal therapeutic modality should go beyond direct cancer cell killing, by indirectly eliciting the amplification of naturally occurring endogenous anti-cancer mechanisms to decrease the stochastic chance of developing therapy-resistant subclonal cancer cell populations.

This principle supports the adoption of therapies affecting the multicellular network of the TME to redirect its predominantly immune suppressive functions toward cancer killing [12]. Since most endogenous anti-cancer mechanisms depend upon the contribution of a combination of innate and adaptive immune cells, this phenomenon is called: “*immune-mediated cancer rejection*”.

### Immune-mediated cancer rejection

Immune-mediated cancer rejection is a facet of the continuum of cancer immune surveillance culminating in immune-mediated tissue-specific destruction (ITD). In simpler terms, cancer rejection equates to an autoimmune reaction against neoplastic tissue [1, 30]. ITD is a conserved evolutionary mechanism meant to protect the survival of the species by safeguarding against infections in animals of reproductive age. Cancer is not a threat to our species since it prevails mostly past the reproductive cycle. Thus, immune reactions against cancer are not an evolutionary requisite but rather represents an epiphenomenon occasionally triggered when cancer cell death mimics infection: a phenomenon referred to as “*immunogenic cell death*” (ICD) [2]. ITD requires the activation of a specific gene signature termed the immunologic constant of rejection (ICR) that stands as an absolute prerequisite for its occurrence [1, 30].

The organism needs to maintain a balance between defending against something that looks foreign and preventing immune-mediated destruction of healthy tissues. However, several innate immunity defense mechanisms are promiscuous and cannot accurately discriminate between pathologically affected cells and surrounding healthy cells. Thus, ITD is counteracted by regulatory mechanisms that balance the destructive power of immune effector cells. As the cause of inflammation is gradually removed by clearing the pathogenic condition, regulatory mechanisms override effector ones and normality is restored. If the cause cannot be cleared completely, a balance is stricken and smoldering chronic inflammatory processes persist. When this balance goes awry in the opposite direction, ITD can lead to destructive flares of autoimmunity, allograft rejection and graft-*vs*-host disease [1, 31].

Applied to cancer, the ICR denotes activation of immune effector mechanisms in the TME such as the Th1 polarization of effector cells, production of immune-stimulatory cytokines such as interferon (IFN)-$\gamma$ and interleukin (IL)-12, and the release of CXCR3- and CCR5-ligand chemokines that further attract immune cells. When present, the Th1 polarized TME is harbinger of better prognosis in several cancers and is an independent predictor of responsiveness to immunotherapies including the systemic administration of IL-2 [32], CPI [33] ACT [34]. However, the presence of the ICR signature alone is not sufficient in most instances to induce spontaneous cancer regression. Thus, the aim of anti-cancer immunotherapy is to facilitate and amplify the potency of this otherwise natural phenomenon [30].

The ICR signature is the hallmark of immune active cancers and is directly correlated to the amount of immune infiltration in highly immune infiltrated cancers [35–37]. However, not all cancers present with this phenotype. Three immune landscapes characterized by different degrees of infiltration and spatial distribution of CD8^+^ T cells are observable across most cancers [9, 10, 20]. Immune desert/cold cancers lack CD8^+^ T cells and are least likely to respond to immunotherapy and the ICR signature is absent. Immune active/hot cancers can be subdivided into immune excluded (CD8^+^ T cells are segregated at the periphery of tumor nests) or immune infiltrated (CD8^+^ T cells are present at the periphery and within the tumor nests) [1, 32, 38]. The latter are most likely to respond to immunotherapy and are characterized by high levels of expression of the ICR gene signature.

Importantly, in immune infiltrated tumors, the ICR signature is always associated with ICD due to the release of damage-associated molecular patterns (DAMPs) by dying cancer cells [19, 39]. DAMPS mimic signals released by dying pathogen-infected cells called pathogen associated molecular patterns (PAMPs) that initiate the immune response [2, 28, 40–45].

Together with the ICD signature, a broad range of immune suppressive mechanisms referred to as compensatory immune resistance (CIR) consistently accompanies the immune active phenotype as an evolutionary requirement for the immunogenic neoplastic tissue to survive in the immune competent host [19, 39, 43]. CIR includes the checkpoint cluster, T regulatory and myeloid suppressor cells, metabolic inhibitors such as indoleamine 2,3-dioxygenase and nitric oxide synthase, and immune suppressive cytokines such as transforming growth factor (TGF)-β [19, 39]. As previously mentioned, in natural conditions, CIR is meant to reduce immune-mediated destruction of healthy tissues during pathogen infection and override the immune effector mechanisms as soon as the pathogen-infected cells are cleared. This protective mechanism is co-opted by the cancer and allows its survival.

Thus, at steady state, CIR overpowers immune effector mechanisms resulting in gradual tumor growth [19, 30, 39] and this is why even immunogenic tumors can survive and thrive in the immune competent host. Direct or indirect biological approaches to MCN medicines aim at following and amplifying the blueprint of nature, redirecting the multicellular network to tip the balance in favor of immune effector mechanisms (Fig. 1B and C) [1, 12].

### Attuning the TME to immune responsiveness; the “*4 Pillars of success*”.

Therapeutics that target the multicellular network and spark a chain reaction of immune processes that attack cancer cells from different fronts can exponentially decrease the chance of developing treatment resistance. The goal is to achieve complete responses like those observed during immunotherapy that are associated with long-term survival in advanced stage cancers [46].

Gatekeepers to successful immune-mediated cancer rejection are four requirements: (1) presence [9, 10] of T cells in the case of cold tumors since several factors may hamper their ability to traffic and home at the tumor site [49] (2) penetrance of immune effector cells in the TME [20, 50] in the case of immune excluded tumors where T cells are incapable of overcoming either mechanical or functional barriers [20], (3) persistence to ensure that immune effector cells can proliferate at the tumor site and perform their function [51] and (4) predominance over a multitude of immune suppressive [19] and metabolically unfavorable conditions [52]. These requirements are all necessary but insufficient alone and therapies will be successful only if they are met contemporaneously (Table 1).

**Table 1:**
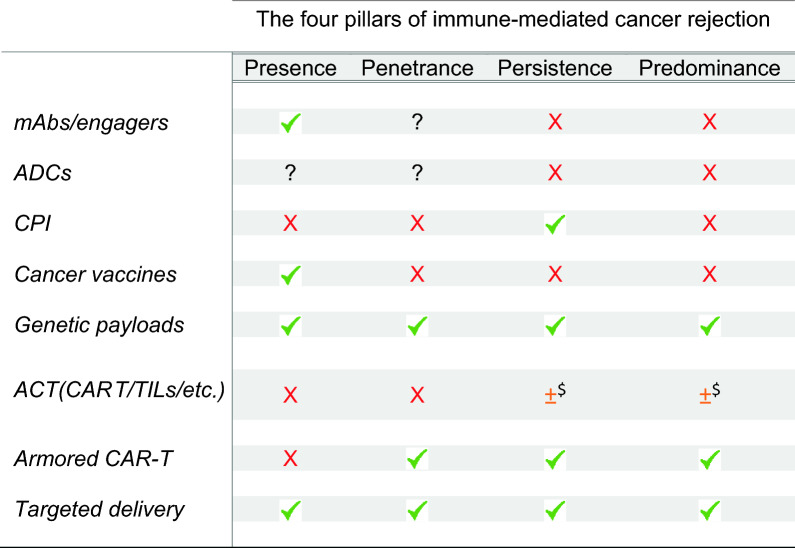
Primary aim of different therapeutics addressing the four pillars of successful immune-mediated cancer rejection

Scoring based on technical assessment of the functional potential of an optimized product

? Conditional effect beyond direct scope of therapeutic determined by the induction of pro-inflammatory signals by dying cancer cells (Example is in Fig. 1* D*); ^$^ Depending upon quality of product before administration and favorable TME conditions

mAbs/engagers include multi-specific mAbs including and CD3-based T cell engagers and similar. ADC = antibody drug conjugates

Genetic payloads include methods for delivery of genetic material short of cellular product or molecularly engineered nanoparticles

Basic CAR Cell (CAR-carrying cell products) with no enhancements based on modern synthetic biology approaches [47]

Armored CAR-T (or other cell products) = next generation CAR-T cells or other cell products that may include conditional and reversible gene activation, logic gating for accurate tumor antigen recognition [47]

Targeted delivery = nanoparticles molecularly engineered to deliver anti-tumoral payloads[48]

The four pillars of success represent concepts aligned with specific gene signatures corresponding to precise functional implications included in the ICR signature [1, 53]. Presence is determined by chemo attractive signals determined by the expression of CCR5 and CXCR3 ligand chemokines. Penetrance into the TME is dependent upon the elimination of chemo-repulsive barriers induced by signals like TGF-β that can be overcome by the production of powerful inflammatory cytokines such as IL-12 [54–57]. CAR T cells armored with factors that can reduce chemo-repulsion may be able to overcome the functional or dynamic barriers that exclude them from tumor nests [20]. Examples include dominant negative [58] or switch TGF-β receptors that make CAR T cells resistant to the immune repulsive and suppressive effects of TGF-β [47, 59, 60]. Moreover, epigenetic knockdown of PD1 expression in response to Ag encounter can overcome dynamic barriers dependent upon PD1/programmed death ligand 1 at the periphery of tumor nests [20, 61]. Persistence is dependent upon the production at the tumor site of homeostatic cytokines such as IL-15 [53] and the reduction of anti-proliferative signaling well exemplified by CPI therapy [62] or epigenetic reprogramming of T cells to knock down CPI expression [61]. Finally, preponderance over immune suppression is dependent on the production of inflammatory cytokines that can repolarize the MCN toward a Th1 phenotype [53] best exemplified by the redirection of macrophages from an M2 to an M1 phenotype [12, 63–65].

Current approaches, however, are designed with the intent of addressing one or few requirements at the time, expecting the other ones to concretize on their own; and this accounts for the rarity and capriciousness of successful outcomes. One example is CPI therapy directed against PD1 that primarily focuses on the persistence of T cells in an immune active TME. Its limited efficacy, particularly in specific cancer indications, is due to its dependence on the preexistence of all the other requirements in the targeted tumors; T cells need to be present or able to be recruited within the TME [66], and PD1 has to play a dominant role over other CIR mechanisms [19]. Similarly, most small molecules and ADCs are aimed at direct cancer cell cytotoxicity. Although they have been shown in some cases to induce ICD, therefore inducing an amplification of the immune effectors in the TME, (Fig. 1B), the effects are often modest and insufficient to induce tumor eradication [3–6]. Thus, it is likely that traditional small molecules addressing a single MOA may at best induce activation of chemotaxis through the release of DAMPs to attract immune cells and offer the exposure to Ags, while stronger immune effects leading to persistence and predominance of immune effector cells over CIR mechanisms are unlikely. Bispecific immune cell engagers aimed mainly at recruiting and activating immune effector cells in the TME but have little potential to address other essential requirements [67]. Finally, vaccines are meant to increase the frequency of circulating cancer-specific memory T cells with the secondary benefit of increasing the chances of their localization in the TME; however, they do not address any other requirement.

The application of genetic engineering can be used to tailor therapeutics to all components of the TME that affect the four pillars. A compendium of immune modulation can be fine-tuned to induce regulated cancer cell death while inducing a temporary pre-terminal activation of their transcriptional and/or translational programs to produce immune activating factors. Chemoattraction can be induced by activation of cancer cell intrinsic type I IFN signaling or transgenic production of IFN-$\gamma$. This in turn can promote the presence and penetration of circulating T cells in cold and immune excluded tumors. Persistence of immune cells can be improved by the transgenic delivery of IL-15 [68] or IL-21 [69, 70] to optimize proliferation and persistence of tumor-reactive CD8^+^ memory T cells. Production of pro-inflammatory cytokines such as IL-12 [71] or the IL-1 family members such as IL-18 [72], together with IFN-$\gamma$ can redirect the immune suppressive milieu toward an immune effector polarization. This is exemplified by the M2 to M1 transition of macrophages that elicit macrophage-mediated extracellular killing and potentiate IL-12-mediated Th1 responses [8, 12, 63–65, 71] or by the Th1 polarization of otherwise immune suppressive tumor associated stromal cells [73].

Here, we argue that cancer-specific delivery of complex genetic payloads allows for the design of next generation therapeutics that holistically address the 4 Ps by delivering a sophisticated combination of functions through direct targeting of cancer cells. Among various possibilities, ACT with immune effector cells and nanoparticles delivering genetic payloads seem most promising.

### The Achilles heel of ACT

ACT with TILs, CAR T cells or other immune effector cells follows a hybrid approach; while directly targeting cancer cells, immune effector cells produce powerful immunogenic stimuli by releasing homeostatic and pro-inflammatory cytokines such as IL-2, granulocyte–macrophage colony stimulating factor (GM-CSF), IFN-$\gamma$, and tumor necrosis factor (TNF)-α, while simultaneously inducing the shedding of Ags and DAMPs by dying cancer cells [7] (Fig. 1B). In addition, armoring of immune cells with additional genetically or epigenetically controlled functions can increase their persistence for instance by knocking out or down the expression of PD1 [61, 74]. Indeed, the ability of ACT products to survive and proliferate upon reaching the TME by maintaining a stem cell like phenotype is critical and it has been clearly demonstrated in the context of solid [51, 75, 76] and hematological malignancies [77]. Moreover, overcoming CIR remains the ultimate frontier for all immunotherapies including ACT and this can be attained by building smart ACT products [47] that can safely redirect the multicellular network of cancer toward an ICR-like phenotype through the contextual delivery, limited for safety reasons to the TME, of powerful immunostimulatory factors such as IL-1 family members [78] or IL-12 [8, 61].

However, a fundamental limitation of ACT is its dependence on chemo attractive signals that promote trafficking of ACT products to the TME. Since CAR T cells and TILs, like endogenous T cells, do not spontaneously home to non-inflamed tissues, they do not traffic to cold tumors and suffer from the inability to penetrate immune-excluded ones [9, 20, 50, 79]. Thus, presence and penetrance into the TME in cold or immune excluded tumors that represent approximately two thirds of all cancers is the prevalent barrier to ACT efficacy [9]. As an example, almost three decades ago, Pockaj et al. [80] observed that TILs, labeled with radioactive ^111^Indium to facilitate their tracking in vivo, did not localize to the intended metastatic tumor sites in about half of the patients who received ACT treatment; in the absence of TIL localization none of the patients experienced tumor regression.

Thus, the ability to direct ACT products to the TME remains in our opinion the unanswered challenge for their success while modulation of their persistence and preponderance can be achieved with ever more powerful synthetic biology tools [47].

### What’s next?

New synthetic biology approaches allow for the direct delivery of payloads by cancer-specific, natural or synthetic nanoparticles capable of targeting tumor cell surface antigens [48]. These payloads can induce regulated cell death of cancer cells directly, while simultaneous transgenic expression of immune modifiers (before the death of the same cancer cells) can exponentially activate the relevant players of the MCN in the TME [81–83] (Fig. 1B). With improvements in biodistribution, nanoparticles could overcome the major hurdle suffered by ACT products, which is their localization in the TME. Specificity can be achieved by CAR-like binders that target Ags present on the surface of tumor cells. Thus, these nanoparticles can act as surrogates of CAR T cells, bypassing the complexity of ACT production and thereby representing a more efficacious and cheaper off-the shelf tool. Similar tumor targeting payload delivery systems could also be considered such as protein/peptide-based delivery systems [84, 85], DNA origami [86], oncoviruses [87, 88], and bacteriotherapy [89]. Although each approach has its limitation, new synthetic biology and molecular engineering tools are likely to overcome obstacles related to each modality in the near future.

We argue here that the future of successful anti-cancer therapy directly or indirectly aimed at inducing the activation of the ICR, is based on a deeper understanding of the requirements determining immune-mediated cancer rejection; by following the blueprint of nature exemplified the phenomenon of cancer immunosurveillance [30], future research can be streamlined. As the 4 Ps entail the concerted activation and polarization of the MCN in the TME, future approaches should recapitulate this natural phenomenon, turning immune desert or immune excluded tumors into immune infiltrated ones and polarizing the latter into an immune effector phenotype that can predominate over the multiple immune suppressive mechanisms present in the TME [19].

In summary, the recipe for effective cancer cure should 1) induce direct cancer killing, 2) be capable to initiate a chain reaction of chemo-attraction that attract immune cells to the TME, 3) enhance Ags shedding to prime memory T cell responses, 4) produce homeostatic cytokines to promote the persistence and proliferation of immune effector cells, and 5) produce pro-inflammatory factors that turn smoldering chronic inflammation into an acute, immune-mediated, tissue-specific destructive process that induces full activation of the ICR signature.

## Abbreviations

ACT: Adoptive cell therapy
ADC: Antibody–drug conjugate
Ags: Tumor-associated antigens
CAR: Chimeric antigen receptor
CIR: Compensatory immune resistance
CPI: Checkpoint inhibitor therapy
CTLA4: Cytotoxic T-lymphocyte associated protein 4
DAMP: Damage-associated molecular patterns
GM-CSF: Granulocyte–macrophage colony stimulating factor
ICD: Immunogenic cell death
ICR: Immunologic constant of rejection
IFN: Interferon
IL: Interleukin
ITD: Immune-mediated tissue-specific destruction
MCN: Multi-cellular network
MOA: Mechanism of action
PAMP: Pathogen-associated molecular patterns
PD1: Programmed cell death protein 1
SIRPα: Signal regulatory protein α
TGF: Transforming growth factor
TIL: Tumor infiltrating lymphocyte
TME: Tumor microenvironment
TNF: Tumor necrosis factor
